# *FBXW7* missense mutation: a novel negative prognostic factor in metastatic colorectal adenocarcinoma

**DOI:** 10.18632/oncotarget.16848

**Published:** 2017-04-05

**Authors:** Krittiya Korphaisarn, Van Karlyle Morris, Michael J. Overman, David R. Fogelman, Bryan K. Kee, Raghav Kanwal Pratap Singh, Shanequa Manuel, Imad Shureiqi, Robert A. Wolff, Cathy Eng, David Menter, Stanley R. Hamilton, Scott Kopetz, Arvind Dasari

**Affiliations:** ^1^ Department of Gastrointestinal Medical Oncology, The University of Texas MD Anderson Cancer Center, Houston, TX, USA; ^2^ Division of Medical Oncology, Department of Medicine, Faculty of Medicine, Siriraj Hospital, Bangkok, Thailand; ^3^ Department of Pathology, The University of Texas MD Anderson Cancer Center, Houston, TX, USA

**Keywords:** colorectal cancer, *FBXW7*, mutation, missense, prognosis

## Abstract

**Background:**

*FBXW7* functions as a ubiquitin ligase tagging multiple dominant oncogenic proteins and commonly mutates in colorectal cancer. Data suggest missense mutations lead to greater loss of *FBXW7* function than other gene aberrations do. However, the clinicopathologic factors and outcomes associated with *FBXW7* missense mutations in metastatic colorectal cancer (mCRC) have not been described.

**Methods:**

Data were obtained from mCRC patients whose tumors were evaluated by next-generation sequencing for hotspot mutations at The University of Texas MD Anderson Cancer Center. Alterations in *FBXW7* were identified, and their associations with clinicopathologic features and overall survival (OS) were evaluated.

**Results:**

Of 855 mCRC patients, 571 had data on *FBXW7* status; 43 (7.5%) had *FBXW7* mutations, including 37 with missense mutations. R465C mutations in exon 9 were the most common missense mutations (18.6%). *PIK3CA* mutations were associated with *FBXW7* missense mutations (p=0.012). On univariate analysis, patients with *FBXW7* missense mutations had significantly worse OS (median 28.7 mo) than those with wild-type *FBXW7* (median 46.6 mo; p=0.003). On multivariate analysis including other known prognostic factors such as *BRAF* mutations, *FBXW7* missense mutations were the strongest negative prognostic factor for OS (hazard ratio 2.0; p=0.003).

**Conclusions:**

In the largest clinical dataset of mCRC to date, *FBXW7* missense mutations showed a strong negative prognostic association.

## INTRODUCTION

Colorectal cancer (CRC) is the third most common cancer worldwide and the second most common cause of cancer-related mortality. It has been well established that colorectal tumorigenesis is a multistep process with an accumulation of multiple, successive genetic alterations, including chromosomal abnormalities, gene mutations, and/or epigenetic changes transforming normal colonic epithelium to colorectal carcinoma [1]. *APC*, *TP53*, *RAS*, *RAF*, and *PIK3CA* gene mutations are the most commonly noted aberrations in metastatic CRC (mCRC). While the prognostic and predictive implications of a few such aberrations, including those of *RAF*, *RAS*, and deficient mismatch repair (MMR), are well established in CRC and now routinely assessed as part of clinical care [2–4], the clinical implications of other genetic aberrations in CRC are unclear in spite of extensive work over the past few decades. Intense research efforts are ongoing to identify reliable, novel biomarkers to help clinicians make personalized treatment decisions in CRC.

A potential avenue to this end involves ubiquitin-mediated proteolysis, which regulates the degradation of many proteins involved in the control of cell growth and differentiation. F-box proteins, the substrate-recognition subunit of SKP1–Cullin1–F-box protein ubiquitin E3 ligase complexes, have been well characterized and shown to play important roles in degradation of proteins regulating cell cycle progression. So far, more than 70 putative F-box proteins have been identified in the human genome; however, the function and the substrates of most F-box proteins remain elusive [5].

*FBXW7* is a tumor suppressor gene on human chromosome 4q that encodes the substrate recognition components of SKP1–Cullin1–F-box protein ubiquitin E3 ligase complexes [6]. These specific E3 ligase complexes negatively regulate the intracellular abundance of an expanding list of key oncogenic proteins such as cyclin E [7], c-JUN [8, 9], c-MYC [10, 11], MCL1 (myeloid cell leukemia 1) [12, 13], NOTCH [14–17], AURKA (aurora kinase A) [18, 19], KLF5 (Krüppel-like factor 5) [20], mTOR [21], and TGIF1 [22]. Therefore, the loss of *FBXW7* function results in accumulation of its substrates, which leads to oncogenesis and progression of multiple cancers including CRC [23, 24]. A study of over 500 primary tumors of diverse tissue origins suggested that *FBXW7* mutations occurred in approximately 6% of all evaluated tumors. Of these, the most commonly affected tumors were cholangiocarcinoma (35%), (T-cell acute lymphocytic leukemia, 31%), endometrial cancer (9%), and gastric cancer (6%) [24]. *FBXW7* has also consistently been identified as one of the most commonly mutated genes in CRC [25], observed in 6% to 10% of all cases [24–27].

*FBXW7* is structurally composed of several conserved protein-protein interaction domains, including the 40–amino acid F-box, which recruits the SKP1–Cullin1–F-box complex; eight WD40 repeats that bind to substrates; and the D domain located just before the F-box, which facilitates dimerization of *FBXW7*. WD40 repeats 3 and 4 contain three highly conserved arginine residues that play a key role in binding to phosphothreonine residues on substrates, while residues within the other WD40 repeats contribute incrementally [28–31]. The *FBXW7* mutational range is rather atypical; over 70% are missense point mutations affecting amino acids within substrate-binding sites, and two of the key arginine residues described above (Arg^465^ and Arg^479^) are mutational hotspots [5]. The rest are mostly nonsense mutations leading to premature termination of translation of *FBXW7*, while the loss of an entire allele is very rarely noted [24]. Tumor-derived *FBXW7* alleles show a very strong predisposition for missense mutations over nonsense mutations. In fact, some tumor types such as T-cell acute lymphoblastic leukemia have only *FBXW7* missense mutations. This marked skew toward full-length *FBXW7* mutations with impaired substrate binding, instead of prematurely terminating mutants (due to nonsense mutations), is thought to be due to the former group's ability to act as more potent dominant negatives negating the impact of the wild-type protein in *FBXW7* dimers [5].

However, to date, the prognostic significance of *FBXW7* missense mutations in CRC remains to be elucidated. In the current study, we extensively evaluated the clinicopathologic and molecular characteristics of *FBXW7* missense mutations in mCRC and the associated survival outcomes.

## RESULTS

A total of 855 mCRC patients were included in the Assessment of Targeted Therapies Against Colorectal Cancer program at The University of Texas MD Anderson Cancer Center between February 13, 2009, and November 18, 2015. Of these, we identified 571 patients for the current study with data available on *FBXW7* status. The median age of the cohort was 55 years (range 20-82 years), and the ratio of males to females was 1.27. The majority of primary tumors were left-sided colon tumors (243 patients, 42.6%), followed by right-sided colon tumors (195 patients, 34.2%), and the rest were rectal tumors (128 patients, 22.4%). Patient and tumor characteristics are shown in (Table 1).

**Table 1:** Clinicopathological characteristics of study population, n (%)

Variable	Value	%
**No. of patients**	571	100
**Median age (yr, range)**	55, 20-82	
**Age**		
<50 years	197	34.5
≥50 years	374	65.5
**Sex**		
Female	251	44
Male	320	56
**Race/ethnicity**		
Asian	31	5.4
Black	50	8.8
Hispanic	54	9.5
White	432	75.7
No data	4	0.7
**Primary tumor site**		
Right sided	195	34.2
Left sided	243	42.6
Rectum	128	22.4
No data	5	0.9
**Metastases type**		
Synchronous	368	64.4
Metachronous	203	35.6
**Metastatic site**		
Liver only	51	8.9
Not Limited to Liver	520	91.1
**Differentiated**		
Well	1	0.2
Mod	423	74.1
Poorly	141	24.7
No data	6	1
***KRAS***		
wt	284	49.7
mt	286	50.1
Variant	1	0.2
***NRAS***		
wt	545	95.4
mt	26	4.6
***BRAF***		
wt	524	91.8
mt	47	8.2
***PIK3CA***		
wt	477	83.5
mt	90	15.8
Variant	4	0.7
**MMR status**		
Proficient	395	69.2
Deficient	19	3.3
No data	157	27.5
***FBXW7***		
wt	527	92.3
mt	43	7.5
Variant	1	0.2

wt: wild type, mt: mutation

### Frequency of *KRAS, NRAS, BRAF, PIK3CA*, and *FBXW7* mutations and MSI-H

**Figure 1:**
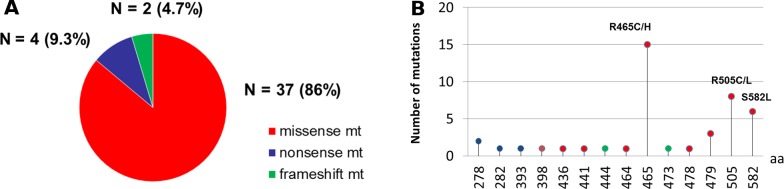
Frequency and spectrum of *FBXW7* mutations **(A)** *FBXW7* mutations were identified in 43 patients. Of these, 37 patients had missense mutations, while four had nonsense mutations, and two had frameshift mutations. **(B)** R465C/H were the most common *FBXW7* missense mutation (n = 15). R505C/L were the second most common *FBXW7* missense mutations (n = 8) followed by S582L missense mutations (n = 6).

DNA was extracted from 363 primary CRC tissues and 208 metastatic tissues. One hundred and ninety four cases were tested with the 46-gene panel while 377 cases were tested with the 50-gene panel. *KRAS*, *NRAS*, *BRAF*, and *PIK3CA* mutations were present in 50.1%, 4.6%, 8.2%, and 16.5%, respectively, of the 571 patients in our cohort. *FBXW7* mutations were identified in 43 patients (7.5%) (Table 1). Of these, 37 patients had missense mutations, while four had nonsense mutations, one had insertion, and one had deletion. R465C in exon 9 affecting Arg^465^ was the most common *FBXW7* missense mutation (18.6%). R465H (affecting Arg^465^) and R505C (affecting Arg^505^) were the second most common *FBXW7* missense mutations (16.3% each). No difference in mutation frequency in *FBXW7* was noted either between primary CRC and metastatic tissues (8.8% vs 5.3%, respectively; P=0.12) (Supplementary Table 1) or between the two gene panels (7.2% vs 7.7%, respectively; p=0.39) (Supplementary Table 2). The frequencies and types of *FBXW7* mutations are shown in (Figure 1 and Supplementary Table 3). MSI-H was found in 3.3% of patients tested (19/414).

### Associations between *FBXW7* mutations and clinicopathologic factors

The clinicopathologic variables compared with *FBXW7* status included age, sex, race/ethnicity, site of the primary tumor, histologic grade, and *KRAS, NRAS, BRAF, PIK3CA*, and microsatellite instability status. Among these factors, only *PIK3CA* mutations were significantly associated with *FBXW7* mutations (p=0.012). A subset analysis limited to *FBXW7* missense mutations showed a similar association between the missense mutations and *PIK3CA* mutations (p=0.022) (Table 2). However, after excluding *PIK3CA* variant mutations (n = 4), there was only a trend towards co-occurrence of *PIK3CA* and *FBXW7* mutations (P=0.10).

**Table 2:** *FBXW7* status and associated clinicopathological factors

Variable	*FBXW7* status							
	wt		All *FBXW7* mutations			*FBXW7* missense mutations		
	N	%	N	%	P value	N	%	P value
**Age (N=570)**								
<50 years	183	34.7	14	32.6	0.77	13	35.1	0.96
≥50 years	344	65.3	29	67.4		24	64.9	
**Sex (N=570)**								
Female	233	44.2	18	41.9	0.77	14	37.8	0.45
Male	294	55.8	25	58.1		23	62.2	
**Race/ethnicity (N=566)**								
Asian	29	5.5	2	4.7	0.99	1	2.7	0.89
Black	46	8.8	4	9.3		3	8.1	
Hispanic	50	9.6	4	9.3		4	10.8	
White	398	76.1	33	76.7		29	78.4	
**Primary tumor site (N=565)**								
Rt.sided	182	34.9	12	27.9	0.42	8	21.6	0.17
Lt.sided	225	43.1	18	41.9		17	45.9	
Rectum	115	22	13	30.2		12	32.4	
**Metastases type**								
Synchronous	341	64.7	26	60.5	0.58	21	56.8	0.33
Metachronous	186	35.3	17	39.5		16	43.2	
**Metastatic site**								
Liver only	46	8.7	5	11.6	0.52	4	10.8	0.67
Not Limited to Liver	481	91.3	38	88.4		33	89.2	
**Differentiated (N=564)**								
Well to moderately	392	45.2	32	74.4	0.91	27	73	0.76
Poorly	129	24.8	11	25.6		10	27	
***KRAS* (N=569)**								
wt	265	50.4	19	44.2	0.44	15	40.5	0.25
mt	261	49.6	24	55.8		22	59.5	
***NRAS* (N=570)**								
wt	503	95.4	41	95.3	1	35	94.6	0.69
mt	24	4.6	2	4.7		2	5.4	
***BRAF* (N-570)**								
wt	485	92	38	88.4	0.39	33	89.2	0.53
mt	42	8	5	11.6		4	10.8	
***PIK3CA* (N=570)**								
wt	446	84.6	30	69.8	0.012	26	70.3	0.022
mt	81	15.4	13	30.2		12	29.7	
**MMR status (N=414)**								
Proficient	365	96.1	30	88.2	0.06	26	89.7	0.13
Deficient	15	3.9	4	11.8		3	10.3	

wt: wild type, mt: mutation

### Survival analysis

The median follow-up time was 30.4 months. At the time of data cut-off (March 1, 2016), there were 266 patients alive and 305 patients who had died.

**Figure 2:**
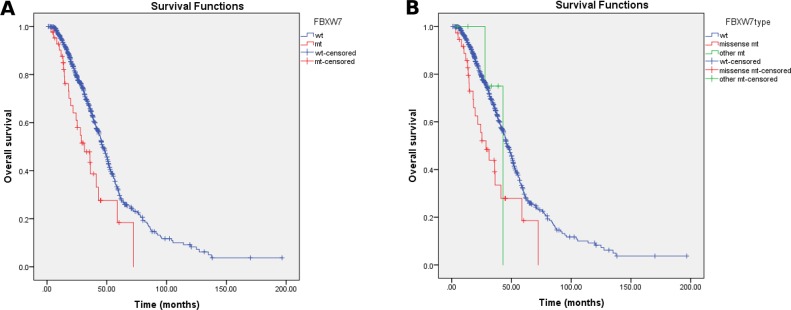
Kaplan-Meier survival curves according to *FBXW7* status **(A)** Patients with *FBXW7* mutations had significantly worse OS (median OS 31.3 mo, 95% confidence interval [CI] 18.7-43.9 mo) than patients with wild-type *FBXW7* (median OS 46.6 mo, 95% CI 43.0-50.1 mo; p = 0.002). **(B)** Patients with *FBXW7* missense mutations had significantly worse OS (median OS 28.7 mo, 95% CI 17.8-39.6 mo) than patients with other *FBXW7* mutations "(median OS 43.0 mo, 95% CI not reached; p = 0.003).

Univariate analysis of OS was performed using previously established prognostic factors: the aforementioned clinicopathologic variables as well as *FBXW7* mutations. Factors that were associated with statistically significantly worse OS in this analysis included an age of <50 years (p=0.016), right-sided tumor origin (p<0.001), poor differentiation (p<0.001), *KRAS* mutations (p<0.001), *BRAF* mutations (p=0.004), and *PIK3CA* mutations (p=0.007). Patients with *FBXW7* mutations had significantly worse OS (median OS 31.3 mo, 95% confidence interval [CI] 18.7-43.9 mo) than patients with wild-type *FBXW7* (median OS 46.6 mo, 95% CI 43.0-50.1 mo; p=0.002 (Table 3, Figure 2A). Patients with *FBXW7* missense mutations had significantly worse OS (median OS 28.7 mo, 95% CI 17.8-39.6 mo) than patients with other *FBXW7* mutations (median OS 43.0 mo, 95% CI not reached (Table 3, Figure 2B). We also looked at *FBXW7*^arg^ and other missense mutations but found no significant associated prognostic effects (Supplementary Figure 1).

**Table 3:** Survival analysis

Variables	N	Univariate analysis			Multivariate analysis		
		Median survival (mo)	95%CI	P value	HR	95%CI	P value
**Age**							
<50 years	197	40.96	34.88-47.05	0.016	1.42	1.10-1.83	0.006
≥50 years	373	47.57	43.58-51.56		Ref		
**Sex**							
Female	251	45.47	41.19-49.75	0.842			
Male	320	45.70	39.61-51.78				
**Site**							
Rt.sided	195	38.20	30.33-46.07	<0.001	1.46	1.03-2.05	0.031
Lt.sided	243	50.27	44.92-55.62		0.98	0.71-1.34	0.891
Rectum	128	48.33	40.44-56.22		Ref		
**Differentiated**							
Well to moderately	424	48.26	44.34-52.19	<0.001	Ref		
Poorly	141	36.49	28.61-44.38		1.52	1.18-1.96	0.001
***KRAS***							
wt	284	50.47	43.62-57.31	<0.001	Ref	1.13-1.91	0.004
mt	286	41.03	35.41-46.65		1.47		
***NRAS***							
wt	545	45.67	42.28-49.06	0.163			
mt	26	37.18	29.37-45.00				
***BRAF***							
wt	524	46.55	42.72-50.39	0.004	Ref		
mt	47	37.71	16.55-58.87		1.72	1.11-2.67	0.015
***PIK3CA***							
wt	477	47.24	43.55-50.94	0.007	Ref		
mt	94	40.73	31.54-49.93		1.12	0.83-1.52	0.451
**MMR status**							
Proficient	395	45.67	40.82-50.51	0.073	Ref		
Deficient	19	35.93	14.76-57.10		1.53	0.72-3.24	0.270
Unknown	157	45.70	39.95-51.45		1.13	0.88-1.45	0.352
***FBXW7***							
wt	527	46.55	43.00-50.11	0.003	Ref		
Missense mt	37	28.67	17.76-39.58		2.0	1.27-3.16	0.003
Other mt	6	42.97	Not reached		0.98	0.24-4.08	0.980

wt: wild type, mt: mutation, Ref: reference

**Figure 3:**
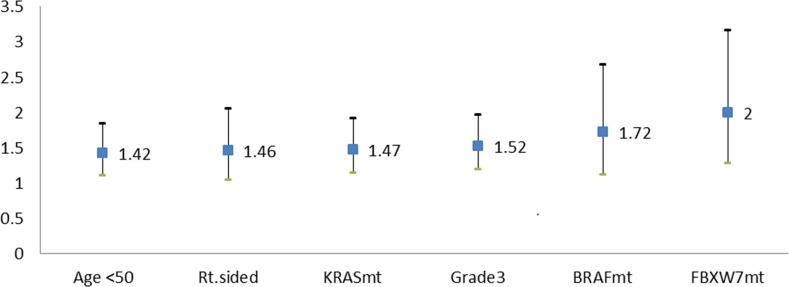
Negative prognostic factors for OS rate (HR with 95% CI) Factors associated with worse OS were an age of <50 years (HR 1.42), right-sided tumor origin (HR 1.46), KRAS mutations (HR 1.47), poor differentiation (HR 1.52), *BRAF* mutations (HR 1.72), and *FBXW7* mutations (HR=2).

Multivariate Cox proportional hazards regression analysis of OS was performed using the factors mentioned above. In this analysis, *FBXW7* missense mutations emerged as the strongest negative prognostic factor for OS (hazard ratio [HR] 2, 95% CI 1.3-3.2; p=0.003). Other factors associated with worse OS were an age of <50 years (HR 1.4, 95% CI 1.1-1.8; p=0.006), right-sided tumor origin (HR 1.5, 95% CI 1.0-2.1; p=0.031), poor differentiation (HR 1.5, 95% CI 1.2-2.0; p=0.001), *KRAS* mutations (HR 1.5, 95% CI 1.1-1.9; p=0.004), and *BRAF* mutations (HR 1.7, 95% CI 1.1-2.7; p=0.015) (Table 3, Figure 3).

## DISCUSSION

In this large cohort of mCRC patients, those who harbored *FBXW7* mutations had significantly worse survival. We showed for the first time that *FBXW7* missense mutations portend a worse prognosis in this population than do other type of mutations

In CRC, the frequency of *FBXW7* mutations has been shown to vary between 6% and 10% and is consistently one of the most frequently mutated genes [24–27]. In our study, the frequency of *FBXW7* mutations was 7.5%, which is consistent with prior studies. In a study by Akhoondi et al. [24] using 1556 samples from a wide range of cancers, including 523 CRC samples, the most frequently noted mutations were single-nucleotide changes, commonly missense mutations, of which nearly half (43%) occurred at two mutation hotspots positioned at codons Agr^465^ (29%) and Arg^479^ (14%). Other mutation hotspots detected in that study were at Ser^582^(4%), Arg^278^ (4%), Arg^224^ (3%), and Arg^393^ (3%). Jardim et al. [32] reported similar results in which the most common mutations were in two hot spots at codons Arg^465^ and Arg^479^.

In our study, most of the detected *FBXW7* mutations were missense (37/43, 86%). *FBXW7* mutations were most commonly found in codon Arg^465^ (15 cases, 34.9%) and were observed in codon Arg^479^in only three cases (7%). Interestingly, our study had a slightly higher frequency of *FBXW7* mutations in codon Arg^505^ (eight cases, 18.6%) than previously described [24, 32]. S582L was noted in six cases (14%) and was the third most common *FBXW7* mutation in our study. Chang et al. [33] reported a similar frequency of *FBXW7* mutations (7.5%, 114/1519) in CRC patients but noted that Ser^582^ (S582L) was the most frequent type (19.3%), ahead of Arg^465^ (R465H, 16.6%), Arg^505^ (R505C, 14.9%), and Arg^479^ (R479Q, 14.9%). These variations are slight and within the realm of inter-study statistical variations. Furthermore, data from the Cancer Genome Atlas Network [34] project confirm that missense mutations affecting the hotspot arginine residues are the most common types in CRC, validating our findings.

*FBXW7* missense mutations, especially those that are *FBXW7*^Arg^ mutations, have a much more profound impact on substrate binding activity than other *FBXW7* gene aberrations because these residues make up a critical substrate-binding interface, and thus disrupt substrate binding [29, 30]. Most of the remaining *FBXW7* mutations are nonsense codons that lead to premature termination of *FBXW7* translation that largely leads to production of non-functional alleles that may or may not dimerize. In contrast, full-length missense mutations result in alleles with dominant negative activity. Multiple mechanisms have been proposed for the more profound effects of missense mutations, including dominant negative effects on wild-type *FBXW7* in dimers by retaining partial activity and stably binding to substrates, preventing their accessibility to wild-type *FBXW7* monomers. Overall, these data suggest that missense mutations in a single allele of *FBXW7* impair the activity of *FBXW7* more profoundly than either allelic loss or stop codons. These data were confirmed in a CRC mouse model with *FBXW7*^Arg/+^ that showed increased tumorigenesis compared with *FBXW7*^+/−^mice. In this model, downstream *FBXW7* substrates were elevated in *FBXW7*^Arg/+^ but not in *FBXW7*^+/−^ tumors [35].

In our study, multivariate analysis demonstrated that *FBXW7* missense mutations were independently associated with poor OS in mCRC and in fact were the strongest negative prognostic factor (HR 2.0) (Figure 3). In particular, *FBXW7* missense mutations had a stronger prognostic association with OS than all *FBXW7* mt or non-missense mutations. This finding confirms our hypothesis that *FBXW7* mutation type has prognostic implications. Given the small numbers in the current study, these results will need to be confirmed in larger datasets.

In contrast to our study, several other CRC studies evaluating the prognostic effects of *FBXW7* mutations have shown varying and even conflicting results from ours. In a study of 1,519 patients with CRC at all stages, Chang et al. [33] showed that *FBXW7* mutations did not impact prognosis. In fact, in that study, subgroup analyses of *FBXW7* mutations showed that R465H, R465C, and R479Q were associated with better 5-year OS rates than other *FBXW7* mutation types were (76.9% vs 56.0%; p=0.012). Similarly, in the VICTOR trial report, *FBXW7* mutations were not associated with disease-free survival in stage II/III CRC [36]. Finally, data from The Cancer Genome Atlas suggest that *FBXW7* mutations were not associated with overall survival. How do we explain these disparate findings? One obvious explanation would be the difference in patient populations. All these datasets included predominantly early-stage disease, while our study was limited to patients with metastatic disease. The differential prognostic effect of a biomarker across advancing stages of CRC is a well-established concept. For instance, *BRAF* gene mutations are a known strong negative prognostic maker for stage IV CRC [2, 37] but not stage II-III CRC [38]. Similarly, MSI-H has positive prognostic effects in early-stage CRC but little if any in stage IV CRC [4]. Secondly, these studies looked at different end points, ranging from disease-free survival to OS. Finally, as demonstrated in multiple studies [29, 30, 35] missense mutations have much more profound implications than other mutations do, which was not specifically addressed in the prior studies. Future studies should be designed to specifically evaluate the role of missense mutations, especially in the metastatic setting.

Our study demonstrated the co-occurrence of *FBXW7* mutations with *PIK3CA* mutations (p=0.012). However, after excluding *PIK3CA* variants in codon I391M which could be potential germline polymorphisms [39] (4 cases), there was only a tendency towards co-occurrence of these two mutations (P=0.10). The TCGA dataset [34] from 212 sequenced CRC cases suggests that *FBXW7* mutations co-occur with *BRAF* mutations (p=0.03) and also have a tendency towards co-occurrence with *PIK3CA* mutations (p=0.25). It should be noted that TCGA dataset did not include *PIK3CA* I391M variants in their analysis. Alternatively, this difference might also be related to the difference in study populations with our study including only advanced stage of tumor while TCGA dataset included all stages with only 29/212 (13.7%) being stage IV. The correlation between *FBXW7* mutations with other gene mutations across different stages needs to be validated in future studies. Lupini et al. reported that *FBXW7* mutations were associated with resistance to anti-epidermal growth factor receptor antibodies (anti-EGFRab). The ability to predict response to anti-EGFRab in *KRAS* WT patients was significantly improved by adding *FBXW7* to a *NRAS-BRAF-PIK3CA* mutation panel (P=0.016 and 0.045 respectively). In this study, the prevalence of *FBXW7* mt was 17.8% (5/28 cases) and 2.7% (1/37 cases) in the non-responder and responder groups, respectively (P=0.08). [40]. However, this is a small dataset and requires further larger studies to confirm this effect.

An obvious direction of future work based on the current findings would be *FBXW7*-directed therapy. Directly, targeting *FBXW7* mutations may be challenging given the disparate spectrum of changes noted and as yet unclear implications of the various aberrations. An easier and more direct approach might be targeting the downstream oncogenic substrates of *FBXW7*. Since *FBXW7* has multiple substrates, it would be important to identify the most important ones in mCRC. Aydin et al. [41] reported that *FBXW7* mutational inactivation represents a mechanism for NOTCH1 activation in melanoma and that anti-NOTCH treatment strategies showed promise in reduction of tumor growth in a xenograft model, making the NOTCH pathway a compelling target for therapeutic intervention. Similar findings have been noted in T-cell acute lymphocytic leukemia as well [42]. Intriguingly, a study by Sancho et al. in APC^min/+^mouse models lacking *FBXW7* activity in the intestine showed more aggressive adenomatous polyposis coli–mediated tumorigenesis and progression. These tumors were noted to have an abundance of Notch pathway substrates. These findings were confirmed in a small set of human CRC samples (five tumor and six control samples) that confirmed increased Notch activity, suggesting that a failure to antagonize Notch activity is an evolutionarily conserved mechanism of loss of function of *FBXW7* [43]. Whether the Notch pathway is truly activated in CRC patients with *FBXW7* mutations and whether the pathway would serve as a predictive biomarker for Notch pathway inhibitors will need to be evaluated.

Our study had several limitations. Firstly, it has the inherent drawbacks of a retrospective study. Secondly, our analysis of *FBXW7* function was limited to only hotspot *FBXW7* gene mutations, and therefore it is likely we missed other mechanisms leading to the loss of function of this protein, including other gene aberrations (such as truncation or deletion) or post-translational modifications. However, to the best of our knowledge, this is the first study to report the clinical characteristics and outcomes associated with *FBXW7* missense mutations in mCRC and show the strong negative prognostic effects of such mutations.

In summary, this is the first and largest clinical dataset of *FBXW7* missense mutations which showed negative prognostic impact on survival in mCRC. Since *FBXW7* was found more frequently in CRC and since most *FBXW7* substrates are oncoproteins, further studies are required to identify downstream pathways underlying this worse prognosis and potential therapeutic targets.

## MATERIALS AND METHODS

In this single-institution, retrospective study, medical records of patients with mCRC who were enrolled in the Assessment of Targeted Therapies Against Colorectal Cancer program at The University of Texas MD Anderson Cancer Center between February 13, 2009, and November 18, 2015, were reviewed, and only patients who had next-generation sequencing data available were included in the study. The study protocol was approved by the MD Anderson Cancer Center institutional review board. All patients provided written informed consent for sequencing of their tumors according to institutional guidelines. The primary objective of this study was to determine the prognostic effect of *FBXW7* missense mutations on overall survival (OS); the secondary objectives were to examine associations between *FBXW7* mutations and various clinicopathologic characteristics and to evaluate the prognostic effect of all *FBXW7* mutations, irrespective of type, on OS.

### Clinical characteristics

Demographic information including age, sex, race, primary tumor site, date of diagnosis with stage IV disease, date of last follow-up, and date of death were collected from the review of the medical records. Right-sided colon cancer was defined as cancer in the region from the cecum to the splenic flexure, while left-sided colon cancer was defined as cancer in the region from the descending colon through the sigmoid colon, and the rectum was considered a separate site. Staging was done per the American Joint Committee on Cancer/Union for International Cancer Control TMN staging system (version 7, 2010) [44]. OS was defined as the interval between the date of diagnosis of metastatic disease and the date of death from any cause; patients alive at the time of analysis were censored at their last known follow-up date.

### Molecular characterization

DNA was extracted from paraffin-embedded formalin-fixed tumor tissue. Samples were evaluated by using a next-generation sequencing platform with 46- or 50-gene panels for the detection of frequently reported point mutations in human malignancies in a Clinical Laboratory Improvement Amendments–certified molecular diagnostics laboratory which determined the effective lower limit of detection (analytical sensitivity) for single nucleotide variations to be in the range of 5% (one mutant allele in the background of nineteen wild type alleles) to 10% (one mutant allele in the background of nine wild type alleles). Details of codons and exons tested in *KRAS, NRAS, BRAF, PIK3CA*, and *FBXW7* are shown in (Supplementary Table 4).

### Determination of MMR status by microsatellite instability testing

MMR status was determined by immunohisto-chemical analysis of MMR protein expression or by polymerase chain reaction analysis of microsatellite instability. Deficient MMR was defined as the presence of high-level microsatellite instability on polymerase chain reaction and/or as the loss of MMR protein expression on immunohistochemical analysis. Proficient MMR was defined as the presence of microsatellite stability or low-level microsatellite instability on polymerase chain reaction and/or as the presence of normal MMR protein expression on immunohistochemical analysis.

### Immunohistochemical analysis of MMR expression

Immunoperoxidase stains were performed on sections from formalin-fixed paraffin-embedded tissues with antibodies for the DNA mismatch repair enzymes MLH1, MSH2, MSH6, and PMS2. Staining for MMR proteins was performed using the following primary antibodies: mouse anti-human MLH1 (clone G168-728; Cell Marque Corporation, Rocklin, CA), 1:300 dilution; mouse anti-human MSH2 (clone FE11; Calbiochem/Oncogene Research Products, Cambridge, MA), 1:100 dilution; mouse anti-human MSH6 (clone 44; BD Biosciences, San Jose, CA), 1:300 dilution; and mouse anti-human PMS2 (clone A16-4; BD Biosciences), 1:125 dilution. Loss of MMR protein was defined as the absence of nuclear staining of tumor cells in the presence of positive nuclear staining in normal epithelial cells and lymphocytes. Interpretation of immunohistochemistry for mismatch repair status is shown in (Supplementary Table 5).

### Polymerase chain reaction analysis for microsatellite instability testing

DNA extracted from microdissected paraffin-embedded tumor sections and non-neoplastic tissues was analyzed by a polymerase chain reaction–based method followed by capillary electrophoretic detection. A panel of seven microsatellite markers (BAT25, BAT26, BAT40, D2S123, D5S346, D17S250, and TGFBR2) was evaluated to detect changes in the number of microsatellite repeats in tumor tissue compared with normal tissue. Tumors were classified as MSI-H (> 40% of markers altered), MSI-L (< 40% of markers altered) and MSS (no marker altered).

### Statistical analysis

Patient characteristics were summarized with descriptive statistics. The relationships between clinicopathologic variables and *FBXW7* status were assessed using Pearson's χ2 or Fisher exact test as appropriate. The associations of patient and molecular characteristics with OS were assessed by Kaplan-Meier estimation, log-rank test, and Cox proportional hazards regression models. Median follow-up time was calculated using the reverse Kaplan-Meier method. All tests were two-sided, and p<0.05 was considered statistically significant. Calculations were carried out using SPSS version 23.0 software (IBM Corporation, Armonk, NY).

## SUPPLEMENTARY MATERIALS FIGURES AND TABLES



## Abbreviations

CRC: colorectal cancer
mCRC: metastatic colorectal cancer
OS: overall survival
MMR: mismatch repair
MCL1: myeloid cell leukemia 1
AURKA: Aurora kinase A
KLF5: Krüppel-like factor 5
MSI: Microsattelite instability
anti-EGFRab: anti-epidermal growth factor receptor antibodies
