# Supplementary material for: FBXW7 missense mutation: a novel negative prognostic factor in metastatic colorectal adenocarcinoma
Source: Oncotarget. 2017 Apr 5;8(24):39268–79. doi: 10.18632/oncotarget.16848 (PMC5503612; doi:10.18632/oncotarget.16848)
Supplement: Supplementary file 1 [file oncotarget-08-39268-s001.pdf]

FBXW7 missense mutation: a novel negative prognostic factor in metastatic colorectal adenocarcinoma

SUPPLEMENTARY FIGURE AND TABLES

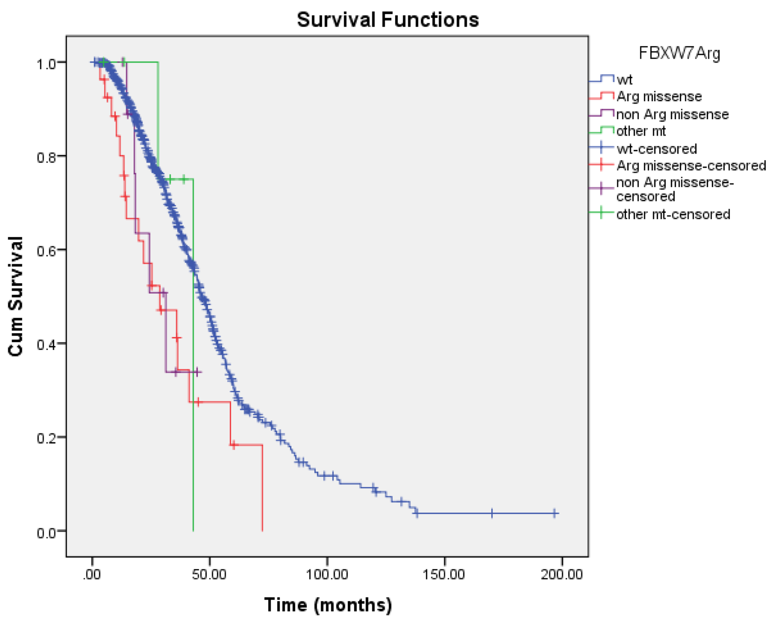

Supplementary Figure 1: Kaplan-Meier survival curve according to *FBXW7* type. Patients with *FBXW7*arg missense mutations had no significant different in OS compared with patients with other *FBXW7* missense mutations(median OS 28.7 mo, 95% CI 10.0-50.1 mo vs 31.3 mo, 95%CI 15.7-46.9 mo).

Supplementary Table 1: Mutation frequency in *FBXW7* on primary CRC and metastatic tissues

		Tissue location		P value
		Primary CRC	Metastatic	
FBXW7	wt	330 (91.2%)	197 (94.7%)	0.12
	mt	32 (8.8%)	11 (5.3%)	
Total		362 (100%)	208 (100%)	

*exclude 1 case that reported variant detection in *FBXW7*

Supplementary Table 2: Mutation frequency in *FBXW7* in 46-, 50- gene panels

Exon	Total case in 46-gene panel (N=194)		Total case in 50-gene panel (N=376)		P value
	N	Mutation frequency	N	Mutation frequency	
**Exon5**					
S282*	0	0/194=0%	1	3/376=0.8%	
R278*	0		2		
**Exon8**					
R393*	0	0/194=0%	1	2/376=0.5%	
S398F	0		1		
**Exon9**					
K444fs*2	0		1		
R473fs*2	0		1		
V464M	0		1		
S436N	0		1		
R441Q	0		1		
R465C	1	6/194=3.1%	7	14/376=3.7%	
R465H	5		2		
**Exon10**					
S478F	0		1		
R505L	1		0		
R479Q	1	6/194=3.1%	2	6/376=1.6%	
R505C	4		3		
**Exon11**					
S582L	2	2/194=1%	4	4/376=1.1%	
	Total=14	14/194=7.2%	Total=29	29/376=7.7%	0.39

Supplementary Table 3: Types and frequencies of *FBXW7* mutations (N=43)

Exon	DNA change	Protein change	N	Total (%)
**Exon5**				
S282*	SNV	Nonsense	1	
R278*	SNV	Nonsense	2	3 (7%)
**Exon8**				
R393*	SNV	Nonsense	1	
S398F	SNV	Missense	1	2 (4.7%)
**Exon9**				
K444fs*2	Insertion	Frameshift	1	
R473fs*2	Deletion	Frameshift	1	
V464M	SNV	Missense	1	
S436N	SNV	Missense	1	
R441Q	SNV	Missense	1	
R465C	SNV	Missense	8	
R465H	SNV	Missense	7	20 (46.5%)
**Exon10**				
S478F	SNV	Missense	1	
R505L	SNV	Missense	1	
R479Q	SNV	Missense	3	
R505C	SNV	Missense	7	12(27.9%)
**Exon11**				
S582L	SNV	Missense	6	6(13.9%)
				43 (100%)

SNV: single nucleotide variant

Supplementary Table 4: Codons evaluated in hotspot mutation analysis

Genes	46-gene somatic mutation analysis panel, Exon(codons)	50-gene somatic mutation analysis panel, Exon(codons)
KRAS	2-3(12,13,19,22,59,61), 4(146)	2-3(5-66), 4(114-150)
NRAS	2(12,13,18),3(61)	2(3-31), 3(43-69), 4(124-150)
BRAF	11(444,464,466,469,471),15(581,586,587, 592,594,595,596, 597,599,600,601, 605)	11(439-473), 15(581-611)
PIK3CA	2(88), 5(345), 8(420), 10(539, 542, 545, 546), 14(701), 21(1021, 1025, 1043, 1047, 1049, 1068)	2(54-90), 2(116-118), 5(316-351), 7-8(390-422), 8(449-468), 10(522-549), 14(677-720), 19(898-924), 21(1017-1051), 21(1065-1069)
FBXW7	5(278),8(393),9(465),10(479,505), 11(582)	5(264-287), 8(378-403), 9(434-473), 10(478-509), 11(567-594),

Supplementary Table 5: Interpretation of immunohistochemical analysis for MMR status

MMR mutation	Immunohistochemical staining			
	MLH-1	MSH-2	MSH-6	PMS-2
MLH-1	-	+	+	-
MSH-2	+	-	-	+
MSH-6	+	+	-	+
PMS-2	+	+	+	-
